# Inspired to Lend a Hand? Attempts to Elicit Prosocial Behavior Through Goal Contagion

**DOI:** 10.3389/fpsyg.2019.00545

**Published:** 2019-03-29

**Authors:** Hilmar Brohmer, Andreas Fauler, Caroline Floto, Ursula Athenstaedt, Gayannée Kedia, Lisa V. Eckerstorfer, Katja Corcoran

**Affiliations:** Social Psychology, Institute for Psychology, Graz, Austria

**Keywords:** goal contagion, goal pursuit, prosocial behavior, social value orientation, social cognition

## Abstract

Helping often occurs in a broader social context. Every day, people observe others who require help, but also others who provide help. Research on goal contagion suggests that observing other people’s goal-directed behavior (like helping) activates the same goal in the observer. Thus, merely observing a prosocial act could inspire people to act on the same goal. This effect should be even stronger, the more the observer’s disposition makes him or her value the goal. In the case of prosocial goals, we looked at the observer’s social value orientation (SVO) as a moderator of the process. In three studies (*N* = 126, *N* = 162, and *N* = 371), we tested the hypothesis that prosocial observations (vs. control) will trigger more subsequent casual prosocial behavior the more the observer is prosocially oriented. In line with the original research, we used texts as stimulus material in Study 1 and short video clips in Study 2 and 3. In Study 1 and 2, SVO was measured directly before the manipulation was induced and in Study 3 even a week prior to the actual experiment. Additionally, we included a second control condition video clip in Study 3, which did not depict human beings. Despite thoroughly developed stimulus material and methods, we found no support for an effect of the interaction, nor of the prosocial observation, but some support for an effect of SVO on casual helping behavior in Study 1 and 2. A mini meta-analysis revealed an effect equivalent to zero for goal contagion and a small, but robust SVO effect across studies. The main implication for the theory of goal contagion is that prosocial goals might not be as contagious as other goals addressed in the literature. We suggest a meta-analytic review of the literature to identify suitable goals and moderators for the goal contagion process.

## Introduction

Human beings are inherently social animals, as they constantly interact with each other and influence each other’s lives in many ways (Dijksterhuis, 2005). We often rely on the prosocial behavior of others—like close kin, friends, or sometimes even strangers—and cannot achieve certain goals if it were not for others lending a hand. Countless examples come to mind, like the mother who supports her daughter with some extra pocket money, or the employee at work who provides help to a coworker finishing a project on time, or the stranger on the street who spontaneously provides help to a grandma crossing the street. But not only the person who receives the help profits from such behavior. Prosocial behavior is essential to both the society and interpersonal relations (e.g., Dovidio et al., 2006, Chapter 8, Tomasello, 2014, Chapters 4 & 5) and is likely innate to humans (Warneken and Tomasello, 2009). Cultures do usually thrive if there is some sort of institutionalized exchange between people who can provide help and people in need for help. However, a prerequisite for those institutions to work on the societal level are functional and positive interpersonal relations built on trust and cooperation among individuals (Cook, 2001; Newton et al., 2018). Because positive interpersonal relations and trust might be fostered by prosocial behavior (Yamagishi et al., 2005), it would be desirable to know how people can get inspired to behave prosocially. One social-cognitive mechanism that might have the potential to facilitate prosocial behavior is goal contagion.

Goal contagion is a process by which a person adopts a goal following a behavioral observation. Thus, people might be motivated to act prosocially and engage in prosocial behavior, because they adopted a prosocial goal after observing others lending a hand. Importantly, goal contagion is distinct from role modeling (Morgenroth et al., 2015) or mimicry (Chartrand and Lakin, 2013), because people could be inspired to pursue a *similar* goal but use *different* goal-directed behavior as the observed person. Furthermore, goal contagion occurs automatically and follows a specific cognitive mechanism.

The underlying principle of goal contagion is based on research on spontaneous causal inferences (Hassin et al., 2002). This framework posits that once we observe another person’s behavior, we automatically try finding causes for her behavior. For example, if somebody helps a coworker to finish a project, we might infer that this person is especially helpful. In this example, we infer a trait to explain the behavior. Additionally, Aarts and colleagues (Aarts et al., 2004; Aarts and Hassin 2005; Hassin et al., 2005) hypothesized that people might also infer goals from behavior, because goals are just another cause for behavior. In one line of experiments, they showed that reading goal-implying sentences (i.e., “The girl buys tools at the DIY shop”) leads to the activation of words and terms in participants that are conceptually related to the goal (i.e., “manual labor”) measured by a word probe task and a lexical decision task (Hassin et al., 2005). This indicates automatic goal inference^1^.

Importantly, in another line of studies, Aarts et al. (2004) extended the previous findings by demonstrating that people, who observed goal-directed behavior, sometimes adopt the same goal. Specifically, they presented heterosexual male participants with the story of a male protagonist buying a female person a drink in a bar, implying the protagonist’s intention to attain the goal of having casual sex with that woman. After reading the story, the heterosexual male participants were asked to provide help in a subsequent task. Results showed that participants who had received the casual sex story were more willing to help a female experimenter compared to those who were asked to help a male experimenter and those who had read a control story. Because being friendly to females could increase the likelihood of intimate relationships, Aarts and colleagues interpreted their male participants’ increased willingness to help as a mean for attaining the automatically inferred goal of having casual sex. Several follow-up studies (Aarts et al., 2005; Dik and Aarts, 2007, 2008; Loersch et al., 2008; Jia et al., 2014; Lee and Shapiro, 2016; Wessler and Hansen, 2016; see also Corcoran et al., 2019) could validate the goal contagion hypothesis in the context of very distinct goals like academic achievements, earning money, or dieting. However, little of this research addresses prosocial behavior as goal to be elicited in observers.

To our knowledge, there is only one article on goal contagion with casual prosocial behavior—understood as helping as it often occurs in everyday life situations—as the focal goal. In these studies, participants viewed short clips about animated objects providing help to other objects (Dik and Aarts, 2007). The helping objects in those animations put different degrees of effort in their helping behavior. The more effort they showed, the more the helping goal was activated in participants and the more participants indicated willingness to help in a subsequent, unrelated task. However, there are goal contagion studies on other forms of prosocial behavior.

Some studies on goal contagion focused on the goal to cooperate (Loersch et al., 2008; Jia et al., 2014). For example, participants observed a team playing football cooperatively and then developed more cooperative strategies within hypothetical sport scenarios (Loersch et al., 2008). Furthermore, there is evidence that other priming techniques could elicit prosocial behavior. These techniques include semantical primes like crossword or scrambled word tasks (e.g., Bargh et al., 2001; Aarts et al., 2005; Prentice and Sheldon, 2015; Sheldon et al., 2015). However, none of these studies measured a prosocial behavior in line with casual helping. Rather, they investigated subsequent cooperative behavior in social dilemmas or a mere cognitive activation.

In the present paper, we want to extend previous findings and examine whether goal contagion does facilitate prosocial behavior. Thereby, we focus on casual prosocial behavior that could be understood as voluntary helping and assisting in situations where neither the benefits of receiving help nor the costs of providing help are very high. In this sense, it is close to helping situations as they can occur on an everyday basis. Casual prosocial behavior does not consume a high amount of resources (like time, money, or energy), but it also does not provide life-changing (or life-saving) assistance. We focus on this type of prosocial behavior for two reasons: first, casual prosocial behavior often occurs in public space and is therefore observable by others and, second, other factors than goal activation that affect prosocial behavior might play a lesser role.

Unlike more severe situations (as when someone is trapped in a burning car), the potential helper should not feel a strong pressure or obligation to help. In addition, situations asking for casual help are usually relatively easy to solve by the person in need him- or herself (as when someone accidentally drops a pile of folders), further minimizing the observer’s feeling of responsibility and pressure by social norms. Although avoiding social punishments or public shaming when the norm of providing help is not followed are certainly concerns that have been rightly addressed in the context of other cooperative and social behavior (Schwartz and Howard, 1984; Fehr and Gächter, 2002; Goette et al., 2006; Rost et al., 2016), we argue that they play only a minor role for casual prosocial behavior. Casual helping also differs from the game-theoretical concept of cooperation which is often based on strategic considerations about individual and mutual payoffs (Axelrod and Hamilton, 1981; Fischbacher et al., 2001; Reuben and Suetens, 2009; Rand et al., 2014; Rand, 2016). Such considerations are less important for casual helping as utilities for outcomes are not as obvious and readily quantifiable. Although they certainly play a role on a more implicit level, they are not as present as in social dilemma games in the lab (e.g., by payoffs for each party in dictator games or points in resource-dilemma games). Finally, casual helping is not much influenced by the helper’s empathic concern regarding the person in need for help. Empathy often increases with the severity of the situation (Darley and Batson, 1973; Cialdini et al., 1997) and is crucial for altruism (Batson et al., 1981, 2007; Leiberg et al., 2011; Tusche et al., 2016). Altruism could result in prosocial behavior that involves sacrificially large costs for the helper. However, in our type of prosocial behavior, which is characterized by low costs not only for the provider for offering help, but for the receiver in case of omitted help, this plays a lesser role.

Taken together, casual prosocial behavior might not be the most dramatic and thought-through type of prosocial behavior. But similar to other acts of kindness (Van Doesum et al., 2013; Van Lange and Van Doesum, 2015), it conveys the message of interpersonal liking and respect. Furthermore, it occurs rather frequently and is often performed in public. Importantly for our research, it is less guided by other factors like empathic concern, the pressure of social norms or strategic considerations. Yet, it definitely provides an opportunity to pursue a prosocial goal. Thus, if goal contagion is a mechanism that can influence prosocial behavior, we are likely to detect it in the context of casual prosocial behavior.

However, prosocial behavior might not be equally contagious for everybody. There is a diverse range of moderators that have been investigated in the literature on goal contagion, such as effort of the observed person (Dik and Aarts, 2007), situational emotions (Jia et al., 2014) or the perceived social distance between the observer and the observed person (Wessler and Hansen, 2016). In the very first study on goal contagion by Aarts et al. (2004), participants’ individual need for money moderated the relationship between the inferred goal (earning money vs. control) and the adoption of that goal (earning money): the goal was most contagious for those in high need for money. This moderation effect suggests that an activation of a goal due to goal contagion is more likely if the goal is previously part of the observer’s general goal system (Kruglanski et al., 2018). Thus, observer-related dispositions could be crucial to be considered in the context of prosocial behavior.

One of the main dispositional factors of prosocial behavior is social value orientation (SVO). SVO indicates to what degree people consider the interest of others in (inter) dependent situations (Messick and McClintock, 1968; McClintock et al., 1973; Kramer et al., 1986; Van Lange, 1999). To measure SVO, participants are confronted with several options of predetermined payoff-allocations and then have to choose between these options (Murphy et al., 2011; for a comparison of different measures, see Murphy and Ackermann, 2014). Eventually, a higher SVO score indicates a greater willingness to benefit an anonymous person rather than oneself^2^. People who would allocate resources in a mutually beneficial way or even provide the anonymous other with more resources are categorized as *prosocials*. On the contrary, people who would rather benefit oneself than another person or even maximize the difference between oneself and another person are categorized as *proselfs*.

SVO is predictive in a wide array of social contexts. It has been demonstrated that the more prosocially oriented people are, the more time they spend on others’ requests (McClintock and Allison, 1989), the more they donate money for charity (Van Lange et al., 2007), the more socially mindful they select preferable objects (Van Doesum et al., 2013), the more often they interpret situations as a cooperative endeavor (Yamagishi et al., 2013), and the more they value moral behavior, including fairness, honesty, and equality (Liebrand et al., 1986; Sattler and Kerr, 1991; Joireman et al., 2003). Prosocials and proselfs differ in their world views resulting in different expectations of others’ behavior (see also *generalized* expectations in Kelley and Stahelski, 1970; Van Lange, 1992; Bogaert et al., 2008; Pletzer et al., 2018). Proselfs think that others would act in a selfish way when given the option to cooperate, whereas prosocials have a stronger initial expectation that others will cooperate in such situations.

In sum, there is ample evidence favoring SVO as a dispositional factor. In that regard, we think that prosocials more than proselfs are stimulated by observing another person helping someone to pursue a prosocial goal themselves. Therefore, we investigate the moderating effect of SVO on the contagion of prosocial goals.

## The Present Research

The main objective of the present research is to test whether observing an act of every day helping facilitates prosocial behavior due to goal contagion and whether this effect depends on the observer’s social value orientation. Specifically, we predict a main effect of goal contagion: participants in the goal condition should adopt the prosocial goal more often than participants in the control condition. However, this effect should be qualified by an interaction with SVO: the goal contagion process should be more pronounced the stronger participants’ are oriented toward prosocial values. In line with previous research, we also expected a main effect of social value orientation in a way that individuals should behave more prosocially with increasing prosocial orientation, regardless of the goal contagion effect.

Three studies were designed to test the aforementioned hypotheses. The first study was conducted as an online study *via* Amazon’s Mechanical Turk (MTurk), the second one was conducted as a lab study with university students, and the third study was again conducted as an online study, but within a population mainly consisting of students. All three studies were conceptually similar and in line with previous research on goal contagion. They differed with respect to the manipulation material (texts in Study 1 and videos in Study 2 and 3), the point in time where SVO was measured (just before the manipulation in Study 1 and 2 and a week before the manipulation in Study 3) and the dependent measure of prosocial behavior. Crucially, the measure of the DV as well as the behavior observed was always consistent with our narrow focus on every day helping as one type of prosocial behavior. For the purpose of transparency, all data and materials are available on the Open Science Framework. Additionally, Study 2 and 3 were preregistered before data collection. Links are provided in corresponding study sections. All studies were approved by the ethics committee by University of Graz and complied with the Declaration of Helsinki.

## Study 1: Contagion of Prosocial Goals in an Online Study

Study 1 was an online study conducted on MTurk. Our first goal was to see if we could identify a goal contagion effect with prosocial behavior. This way, the study was to some degree similar to Dik and Aarts (2007), who also utilized prosocial goals, but additionally manipulated effort. Secondly, we investigated the moderation effect of SVO. We hypothesized that prosocial goals would be more contagious the more prosocially oriented people are. In addition, we expected that prosocial orientation would predict helping behavior in general (McClintock and Allison, 1989).

Similar to the original goal contagion studies (e.g., Aarts et al., 2004), we used short stories where a person was described in a situation where help was required. This happened after the participants’ SVO was assessed and before they were confronted with an unrelated situation aimed at measuring their prosocial behavior. We measured SVO beforehand to avoid having the SVO score being influenced by the manipulation material (see Ackermann et al., 2016).

### Sample and Participants

We determined the required sample size with G*Power (Faul et al., 2007) based on previous effects in the domain of goal contagion. This was problematic because reported effects ranged from small (e.g., Dik and Aarts, 2008, Experiment 3, *r* = 0.16) to large for psychological standards (e.g., Aarts et al., 2004, Study 4, *r* ~ 0.40). Due to small sample sizes in most of the original studies, which potentially introduced a strong bias in the estimates, we reasoned that a medium population effect size (i.e., *r* = 0.3) was most realistic to assume. Therefore, we calculated our sample size for a linear OLS regression model with Δ*R*^2^ representing the effect of the interaction term of SVO and the goal manipulation. For a Δ*R*^2^ = 0.06 (*f^2^* ~ 0.06), α = 0.05, and 1 − β = 0.8, we obtained a sample of *N* = 105 (one-tailed) to 133 (two-tailed) and decided in favor of the larger, more powerful sample.

We collected data from 138 MTurk workers, but had to exclude 12 participants because they either did not score on the dependent variable due to technical issues or failed the attention checks (see Materials and Methods section). The final sample of *N* = 126 had a mean age of 34.2 years of age (*SD* = 11.12), consisted of 49 females, 77 males, and 113 of them were native English speakers. The analysis without the non-native speakers did not change the pattern of results for the main hypothesis, although it did reduce the correlation between SVO and prosocial behavior. An analysis with demographic covariates did not change the pattern either, which is why we decided to report results from the final sample and without covariates below. For interested readers, all other results and analyses, as well as descriptive statistics, can be found online in the OSF^3^.

### Methods and Procedure

**Figure 1:**
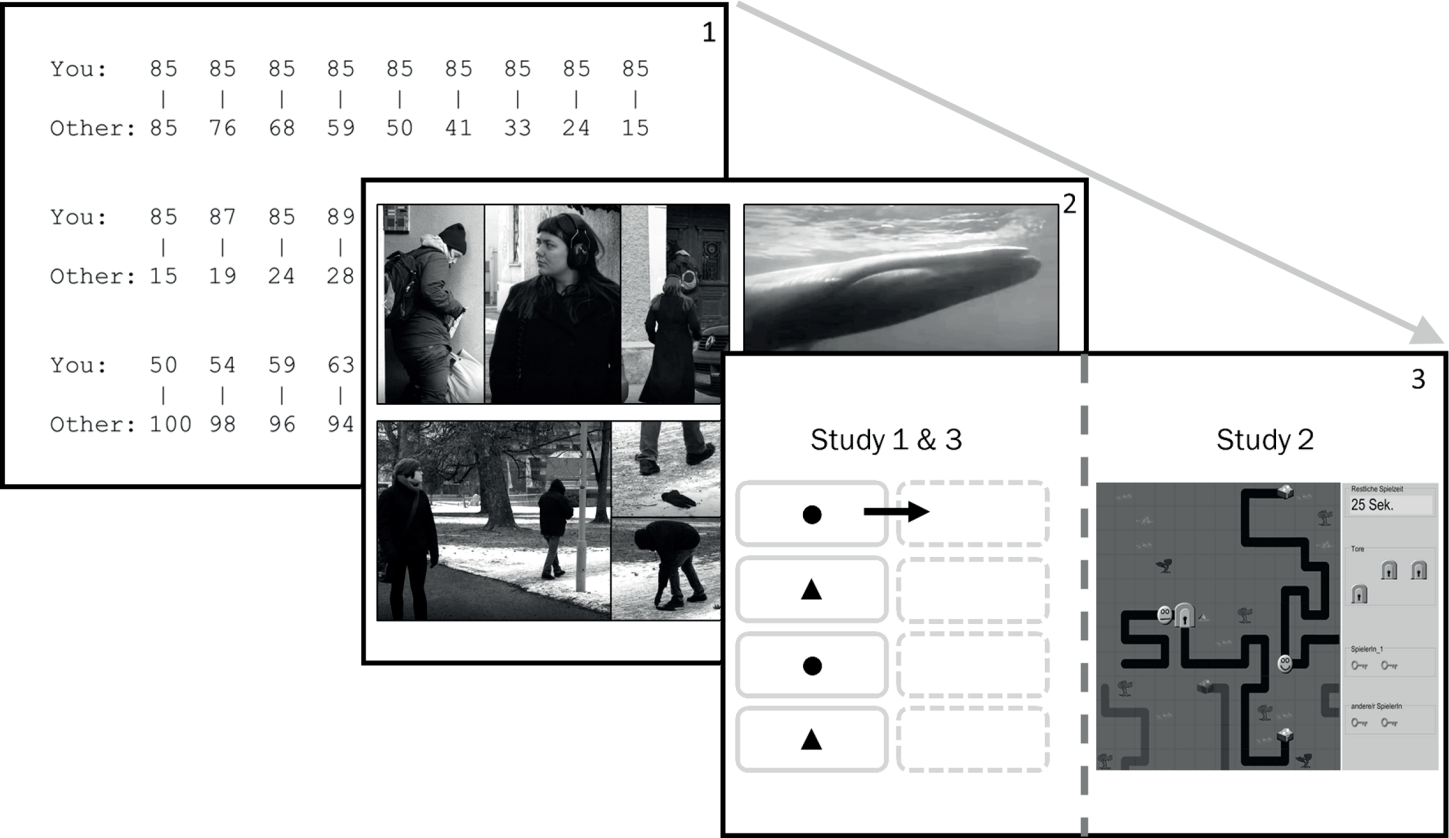
General set-up of Study 1–3. (1) Data on SVO was collected prior to the manipulation (Murphy et al., 2011). (2) Goal manipulation was carried out through texts in Study 1 and video clips in Study 2 and 3. (3) DVs were measured with a drag and drop task in Study 1 and 3, and with the ZPG in Study 2. Note that written informed consent was obtained from all depicted individuals for the publication of these images.

The study was prepared with the survey software Unipark (QuestBack GmbH, 2017). The general set-up of all three studies can be seen in Figure 1. Participants read the consent form and agreed to participate in this 10-min study for $1.00 compensation.

#### SVO

Afterward, they filled out the 6-item SVO measure by Murphy et al. (2011), in which they were instructed to allocate hypothetical resources between themselves and another agent. The resource was represented in “point” units, ranging from 15 to 100 for each person across items. This measure has been demonstrated to have excellent psychometric properties and all participants’ SVOs can be identified and pinpointed on a continuous scale (for details, see Murphy et al., 2011).

#### Goal Manipulation Texts

Subsequently, participants answered demographic questions about their age, ethnicity, education, and gender, the latter of which enabled us to adapt the gender of the characters in the following three short goal manipulation texts (i.e., by adapting pronouns and names). Those three stories were narrated in the third-person perspective and always described a person in an everyday life situation. Story 1 described a person on his or her way to a meeting; in Story 2, the character wanted to catch a connecting train, and in Story 3, he or she was on the bus on his or her way to a date. Hence, in all three stories, the main character had a certain destination just before another person appears in the scenario.

However, the stories differed across experimental conditions: In the goal contagion condition, another character appeared who needed help. In Story 1, the other person accidentally dropped a pile of papers and folders and the main character decided helping him or her to pick up all the folders (vs. control condition in which nothing happened and the main character went on to the meeting). In Story 3, the main character was about to leave the bus and noticed another passenger with a lot of luggage who needs help (vs. control condition in which the other passenger did not have luggage and was not in need of help). Story 2 was a filler story that did not involve any intervention of the main character in either condition. The endings of the stories were always presented on a second page to separate the climax of the story from the conclusion.

The stories were created so that the decision of the main character to help was associated with some small costs (like going on a date, but helping someone heaving luggage or hurrying to a meeting, but assisting someone picking up folders). Moreover, the helping situations we depicted were not severe for the victim. Even if the main character had not offered help, the person in distress would not have been subjected to any harm. We did so to avoid potential emotional confounders in our goal manipulation, which may be typical in related research (Batson et al., 1981, 2007; Cialdini et al., 1997) while highlighting the actual casual helping behavior in an everyday situation.

To ensure that participants had read the texts, we asked them two questions about the content of each story (six in total). Participants who did not answer at least five of those questions correctly were excluded from the analysis (*n* = 10).

#### Volunteering as Dependent Measure

Following the previous questions, participants were partly debriefed. However, on the same page below, they were offered to volunteer in another student’s experiment (similar to Aarts et al., 2004 and Dik and Aarts, 2007 we avoided the word “help” in this description, see OSF). They were told that the student could not compensate them with money because she or he did not receive any funding. However, participants could still support the student by completing some pages of her study. They were also told that they could terminate their participation any time and proceed to the end of the survey. Participants’ agreement or disagreement to start the unpaid extra study was the first dependent measure we obtained (DV1).

The second measure (DV2) was then obtained by the number of pages participants completed in the extra study, described as “Geometrical Figure Task.” Here, they had to drag and drop scrambled symbols, which could vary in color or shape, from the left-hand side of the screen to the right-hand side in a way that similar symbols were stacked next to each other (see Figure 1, third frame). Thus, the task was purposely designed to be neither too complicated nor too exciting in the long run, as too fascinating tasks could confound our DV. After all, we intended to measure prosocial helping rather than excitement over a task. One page could be completed in approximately 10 s and we prepared 160 pages to ensure that participants would be occupied for some time to complete the whole task, if they really intended to finish it. Indeed, we were deliberately unspecific in the instructions about the approximated time to complete the task. This served the purpose that participant’s initial intention to help would not be confounded by their reflection on the length of the task (e.g., if they knew there were 160 pages, they could plan ahead to complete 10 pages only).

Whether Mturk workers participated or not, the last page would contain HIT code for their compensation.

### Results and Discussion

Before submitting the data, we dummy-coded the goal manipulation texts (control texts = 0, goal texts = 1) and centered SVO on the threshold between proselfs and prosocials (SVO = 22.45; see Murphy et al., 2011). The same coding was applied in the subsequent studies. Then, we ran two confirmatory analyses using SPSS 24 with the PROCESS macro (Hayes, 2013). First, we calculated logistic regressions with participants’ decision to help the student (DV1) regressed on SVO and goal manipulation (Model 1). In Model 2, we added the interaction term as predictor. In total, we found that 45 out of 126 (36%) participants chose to help the student in need. However, neither the effect of SVO, Wald’s χ^2^(1) = 2.276, *p* = 0.131, *OR* = 1.021, nor the effect of the goal manipulation, Wald’s χ^2^(1) = −0.032, *p* = 0.931, *OR* = 0.968 led to any significant result. Also, the model with the interaction term (Model 2) did not yield the expected moderation effect either, Wald’s χ^2^(1) = 0.840, *p* = 0.360, *OR* = 0.975, Δχ^2^(1) = 0.846.

**Figure 2:**
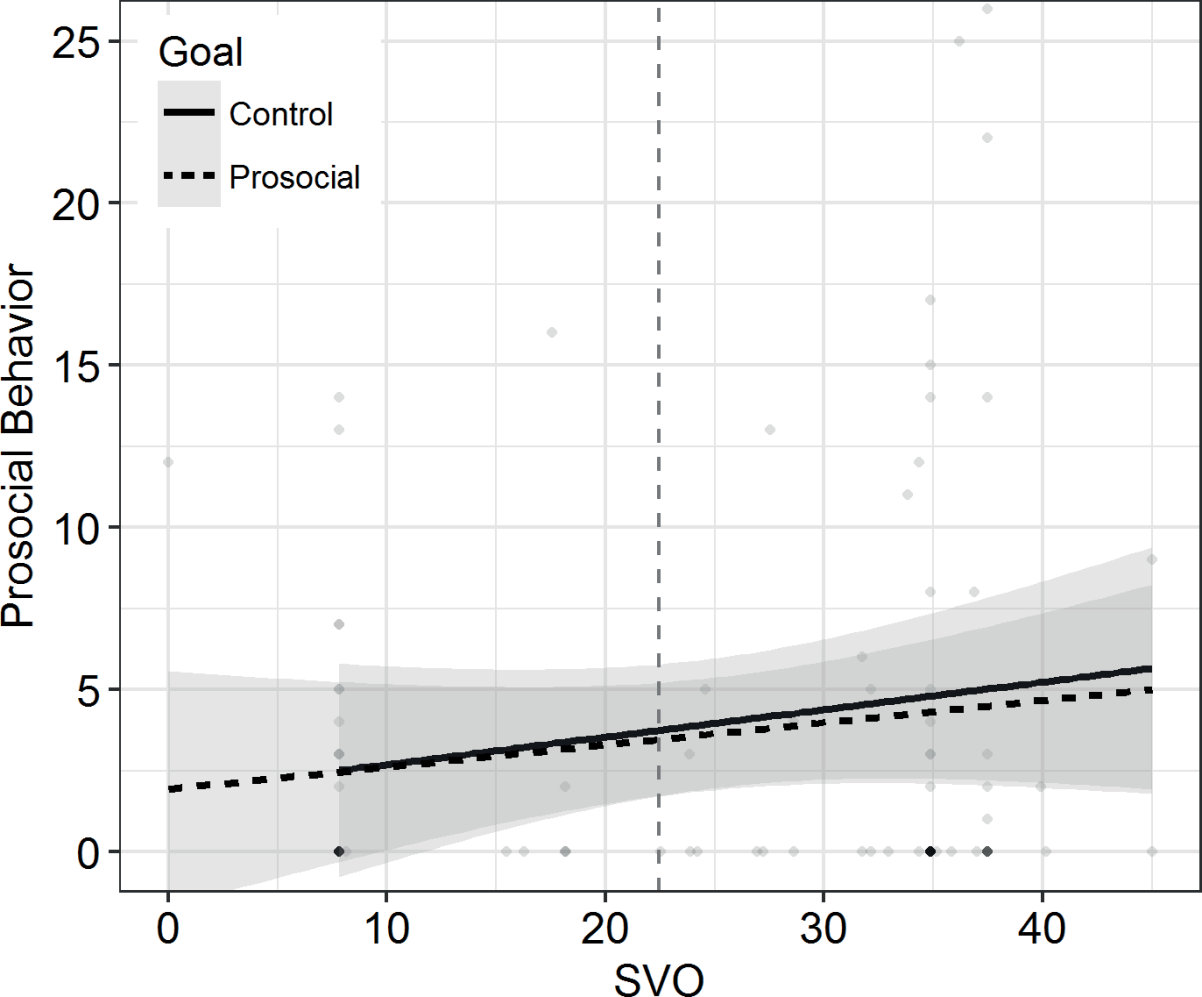
Prosocial Behavior as a function of SVO in Study 1. The separate regression lines represent two conditions. Shades depict 95% CIs. Higher density of the scatter signifies higher concentration of data. Vertical dashed line separates proselfs (left) from prosocials (right).

Second, we regressed the number of completed pages (DV2) on SVO, the goal manipulation (Model 1), and their interaction term (Model 2). As expected, we found a marginally significant effect of SVO in Model 1, *b* = 0.083, *t*(123) = 1.831, *p* = 0.069. However, the goal manipulation did not have an influence on the amount of prosocial behavior provided, *b* = −0.274, *t*(123) = −0.274, *p* = 0.831. Furthermore, the goal manipulation and SVO did not interact, *b* = −0.020, *t*(122) = −0.226, *p* = 0.822, Δ*R*^2^ < 0.001. As visualized in Figure 2, the simple slopes of SVO for both conditions overlapped considerably and did not differ from 0, control condition: *b* = 0.092, *t*(122) = 1.542, *p* = 0.126, prosocial goal condition: *b* = 0.072, *t*(122) = 1.136, *p* = 0.258.

Although we could identify a small effect of SVO, our manipulation for goal contagion did not affect participants’ helping behavior. Importantly, the aforementioned effect of SVO on helping behavior was brought to light only for the continuous dependent measure, but not for the dichotomous measure. This indicates that proselfs and prosocials do not differ much in their mere signaling of providing help, but some differences can be spotted in prosocials’ endurance in providing help.

There may be several reasons why the goal manipulation did not work. First, although several goal contagion studies used text-based priming, these kind of manipulations might be limited in evoking sensitive cognitive processes (see Kanning et al., 2006 for a test of different stimuli). Secondly, MTurk workers might rather be oriented to finish up studies for a financial compensation, as many might even participate for a living (Semuels, 2018). Thirdly, there are few and rather mixed findings on the quality of MTurk studies (Paolacci and Chandler, 2014; Rouse, 2015; Hauser and Schwarz, 2016). Overall, we wanted to address these issues in our second study, which we conducted in a more controlled lab setting in order to get a cleaner effect of goal contagion. Moreover, the lab setting enabled us to utilize another dependent measure suitable for casual helping behavior.

## Study 2: Prosocial Goals in the Lab

In Study 2, we wanted to investigate the contagion of prosocial goals in a lab setting, testing the same hypotheses as before. We intended to use short video clips for our goal manipulation in order to make the helping situation more vivid than in Study 1 and therefore foster the effect. Moreover, we decided to use a relatively recently introduced measure of prosocial behavior: the Zurich Prosocial Game (ZPG; Leiberg et al., 2011). The ZPG can be altered to measure prosocial behavior freed from competitive contexts, contrasting it with other social dilemma games (e.g., Prisoner’s Dilemma, Ultimatum Game or Trust Games). This study, the initial hypothesis, and sample size calculation were preregistered in the OSF before the data were collected^4^.

### Participants and Sample Size

We did not find the expected goal contagion effects in Study 1. Therefore, in Study 2, we aimed for a smaller effect size with a large sample: for Δ*R*^2^ = 0.04 (*f^2^* ~ 0.04) for the relevant interaction effect, with α = 0.05 and 1 − β = 0.8, we obtained a sample of *N* = 156 (one-tailed) to 199 (two-tailed) and decided to collect data from at least 180 participants. Eventually, our sample consisted of 183 students recruited on the campus of the University of Graz, Austria, of which 21 had to be excluded according to the preregistered exclusion criteria (such as missing data on the key variables: *n* = 17; and failed attention checks after the video clips: *n* = 4; see OSF-link above). We were left with a final sample of *N* = 162, with a mean age of 22.60 years (*SD* = 10.50), 101 females, and 160 native German speakers.

Inclusion of demographic covariates did not change the main pattern of results, which is why we focus on the main analysis here. Additional analyses and descriptive statistics can be found in the OSF.

### Methods and Procedure

Participants were welcomed to the lab and seated in front of one of several computer work stations, which were separated by blinds. They filled out the consent form and were introduced to the ZPG (see below) because we wanted to avoid that a later introduction would reduce the effect of the manipulation. SVO, the goal manipulation, and *post hoc* questions were recorded in Unipark (QuestBack GmbH, 2017) and the scores for the dependent measure were recorded in a separate window.

#### SVO

As in Study 1, participants filled out the SVO items by Murphy et al. (2011) prior to the goal manipulation. Afterward they filled out the competitive jungle worldview scale (Perry and Sibley, 2010) for another research question. Inclusion of this worldview as factor in two-way and three-way interactions with the goal manipulation and SVO did not influence the pattern of results (additional variables can be found in the data set, see OSF).

#### Goal Manipulation Video Clips

We produced short video clips depicting three situations in which people showed helpful intentions toward another person in an everyday life situation in the experimental condition (vs. no help was required in the control condition). Similar to the texts from Study 1, the helping situations depicted were not too severe, to avoid inducing empathy in participants.

Situation 1 and 3 differed per experimental condition, whereas Situation 2 was a filler situation and identical in both conditions. In Situation 1, participants saw a woman walking down a narrow street on the sidewalk noticing another person with many grocery bags on the other side of the street, struggling to open a front door. The woman then decides to quickly run to the other side to provide help (vs. someone from inside the building opens the door). In Situation 2, a young woman in a park observes another person’s glove accidently fall out of his pocket while answering a call. However, this other person quickly turns around and picks up the glove. Lastly, Situation 3 depicted a young man in business attire hurrying up a staircase. He passes a female person carrying many folders down the staircase. After she accidentally dropped the folders, he turns around to go back to her (vs. she does not drop the folders and no help is required).

The videos’ lengths were between 24 and 38 s. They were edited in such a way participants would assume helping intentions by the main characters in the experimental condition but not in the control condition. Participants then answered three attention check questions concerning the content of the videos (see the exclusion criteria above).

#### Prosocial Behavior With the Zurich Prosocial Game

In the ZPG (Leiberg et al., 2011; Böckler et al., 2016; Bornemann et al., 2016), participants have to navigate a smiley character along a path that leads to a treasure. Another character—described as another participant, but played by the computer in reality—navigates through a separate path toward his or her own treasure. Obstacle gates fall on both paths randomly and players are required to use keys to open them in order to proceed. We pretested the ZPG and gave detailed instructions to ensure that participants understood the specifics of the game (see OSF).

Our participants were instructed to perform five rounds of this game. To draw participants’ attention toward their treasures, we told them that they would receive a “special reward” (they could draw a lottery ticket from a pot), if they successfully obtained the treasure in at least four out of the five rounds. Importantly, in three of these rounds, we altered the game so that the other character did not have enough keys to open the last remaining gate before arriving at the treasure. The participants always had one spare key that they would not need anymore. Thus, they had the option to help the other player without encountering much cost apart from losing few seconds from their liberal time limit of approximately 50 s (whereby one round was easily completed in 30–40 s). The number of times participants decided to open the other player’s gate counted as the measurement of prosocial behavior. As each participant could offer his or her help in three rounds of the ZPG, the resulting scale ranged from 0 to 3.

The strength of the ZPG lies in the increased subtleness of the experimental procedure compared to other measures. Many established prosociality measures are more overt with respect to their purpose, as participants are relatively explicitly confronted with the situation or asked to provide help (Study 1 & 3 in this paper; Aarts et al., 2004, Study 3; Dik and Aarts, 2007, Experiment 3). Here, the purpose is disguised in the context of a game, where the player’s primary goal is to reach the treasure at the end of a path. Hence, helping the other player in this situation is optional and should depend on whether participants perceive this situation as an opportunity to help through either goal activation or their dispositional SVO.

Before the actual ZPG, participants played two test rounds (before the SVO and the manipulation) aimed at familiarizing them with the task.

#### Post-measure Questions

In the last part, participants were asked demographic questions and several exploratory questions about the manipulation and the ZPG, including three *goal inference* questions about what the goals of the protagonists in the clips had been (for each clip one goal-related answer out of three options) and how often the other player in the ZPG had needed help (0–5 times). We also asked them to what extent they thought that the other player in the ZPG would help them if they were trapped behind a gate (*expectation* measured on a scale from 0 to 100%) and the degree to which they believed that the other player had been another participant (*human player*, 1 “not at all authentic” to 7 “very authentic”). We report those variables for the sake of transparency. Exploratory analyses with those variables can be found in the OSF.

### Results and Discussion

#### Confirmatory Analysis

**Figure 3:**
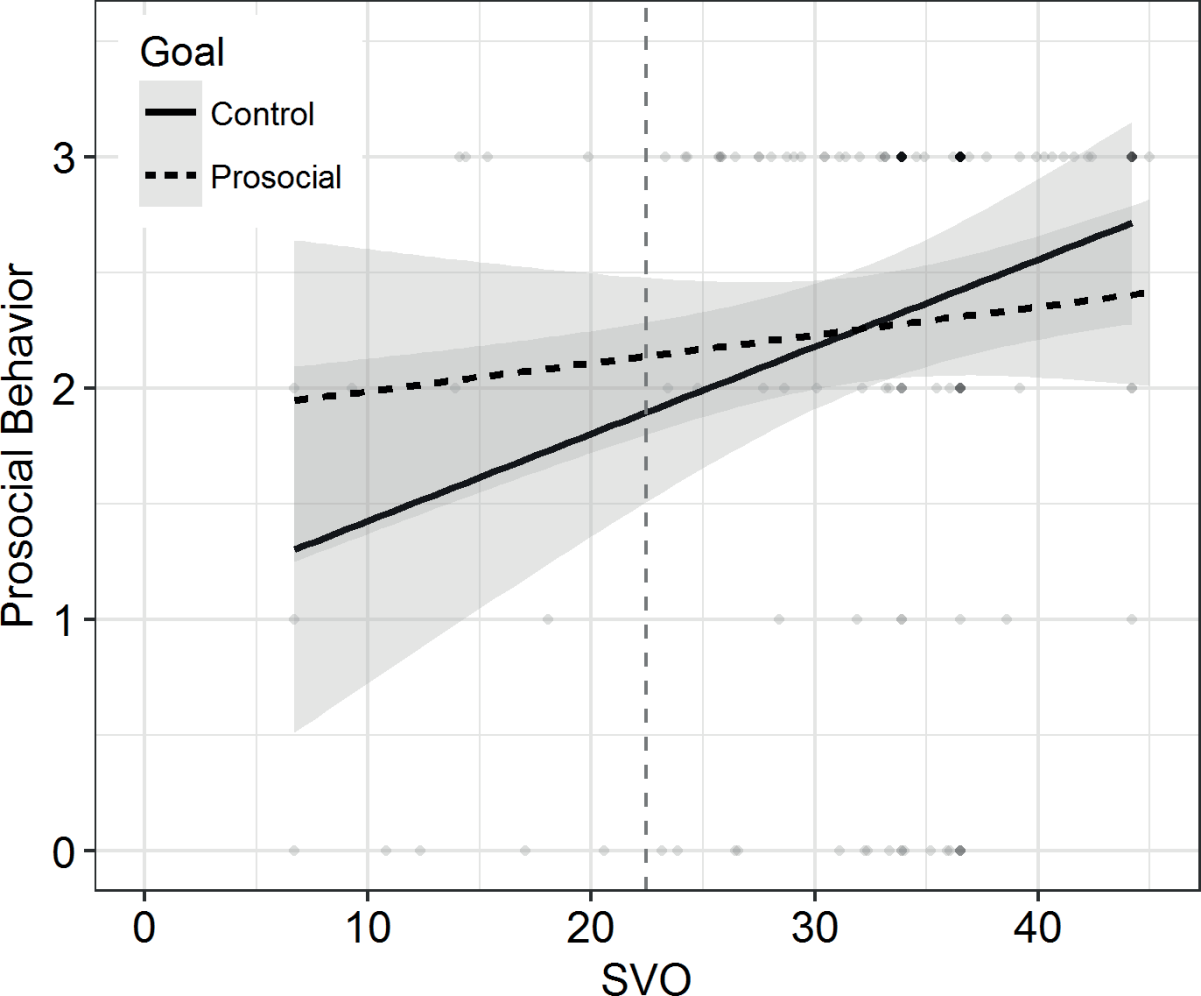
Prosocial Behavior as a function of SVO in Study 2. Separate lines refer to the two conditions. Shades depict 95% CIs. Higher density of the scatter signifies higher concentration of data. Vertical dashed line separates proselfs (left) from prosocials (right).

We computed two OLS regression models. In the first model without the interaction term, we observed an effect of SVO on casual helping, *b* = 0.022, *t*(159) = 2.427, *p* = 0.016, but not of the goal manipulation, *b* = −0.011, *t*(159) = −0.062, *p* = 0.951. Importantly, in the second model, the interaction did not reach significance either, *b* = −0.022, *t*(158) = −1.104, *p* = 0.271, Δ*R*^2^ = 0.009. The simple slope for SVO (see Figure 3) was significant in the control condition, *b* = 0.033, *t*(158) = 2.585, *p* = .011, but not in the goal condition, *b* = 0.011, *t*(158) = 0.846, *p* = 0.399.

#### Exploratory Analyses and Goal Inference as Mediator

As described above, we also collected data on the two variables expectation and human player, which we explored in a regression model with the other predictors. In line with previous research (Pletzer et al., 2018), expectation turned out to be a significant predictor, *b* = 0.027, *t*(155) = 9.601, *p* < 0.001, and the human-player variable was, as well, *b* = −0.082, *t*(155) = −2.094, *p* = 0.038. The latter showed a negative slope coefficient, indicating that the *less* participants believed that the other player was human, the more they behaved prosocially afterward. Therefore, we inspected the item and observed that most participants actually did not believe that the other player was human to begin with, *M* = 2.55, *SD* = 1.74 (on a 7-point scale from 1 “highly inauthentic” to 7 “highly authentic”).

As has been suggested by various studies in the domain of goal contagion (e.g., Aarts et al., 2004), but hardly ever tested (Dik and Aarts, 2008; Jia et al., 2014; Corcoran et al., 2019), it is necessary for a goal to be correctly inferred in order to become contagious. Hence, we suspected *goal inference* to mediate the relationship between the goal manipulation and prosocial behavior. Because we asked participants which goal the people in the three videos pursued, we could build a score of explicit prosocial goal inference. When participants indicated that the people wanted to help somebody, we coded their answer with 1. As a result, participants’ inference scores ranged from 0 (“no inference”) to 3 (“high inference”). Additionally, we kept SVO as a moderator in the model on the direct path between the goal manipulation and prosocial behavior and between goal inference and prosocial behavior.

**Figure 4:**
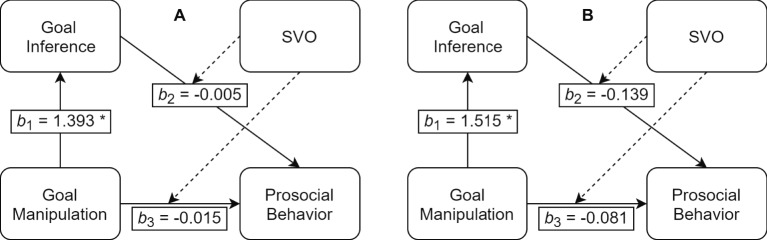
Exploratory mediation models for Study 2 [panel **(A)**] and Study 3 [panel **(B)**]; bs represent unstandardized path coefficients; *b_2_* and *b_3_* are interaction effects with SVO as moderator; note: *p < 0.001.

Therefore, we submitted the data to a mediation analysis with the goal manipulation as IV, prosocial behavior as DV, goal inference as mediator, and SVO as moderator. Goal inference was strongly predicted by the manipulation, *b* = 1.393, *t*(160) = 14.857, *p* < 0.001, but neither goal inference × SVO nor goal manipulation × SVO interactively predicted prosocial behavior, *b* = −0.005, *t*(156) = −0.309, *p* = 0.758 and *b* = −0.015, *t*(156) = −0.536, *p* = 0.593, respectively (see Figure 4). The main effects (based on mean-centered predictors) for SVO, *b* = 0.022, *t*(156) = 2.168, *p* = 0.032, and goal manipulation, *b* = −0.082, *t*(156) = −0.318, *p* = 0.751, had similar slopes as before, and no effect of goal inference on prosocial behavior was spotted, *b* = −0.06, *t*(156) = −0.392, *p* = 0.696. Thus, none of the conditional direct or indirect effects explained prosocial behavior either (see OSF for additional information). This analysis suggests that explicit goal inference did not affect prosocial behavior. Hence, in this study, prosocial behavior could not be attributed to a goal contagion process—neither directly nor over explicit inference.

In sum, results of Study 2 showed a pattern similar to Study 1. Again, prosocial behavior was not influenced by the goal manipulation and goal contagion did not get stronger the more prosocially oriented participants were. However, the prosocial orientation in itself predicted participants’ willingness to help. This relationship underlines that the ZPG indeed assessed variation in prosocial behavior in our study and therefore weakens concern of a null effect due to a fallacious measurement of the DV. Moreover and in line with other research (Aarts, 2004; Jia et al., 2014; see also Corcoran et al., 2019), we could demonstrate an effect of our manipulation on explicit goal inference. However, no relation between goal inference and prosocial behavior emerged. Although participants in the relevant condition had correctly inferred the goal of the observed person, it turned out that this was not a necessary condition for their own prosocial behavior.

The previous two studies did not yield the expected interaction effect of the goal manipulation and SVO, although we found a small but consistent effect of SVO on prosocial behavior. For Study 3, we decided to gain more power by enhancing our priming material further and considerably increase the sample size. Therefore, we decided to turn back to the initial DV measure from Study 1 because that one is more feasible in online settings and because most participants in Study 2 did not believe they were playing against another human player.

## Study 3: Confirming the Effect of SVO

Study 3 aimed at maximizing the power, while addressing some potential critiques of our IVs. First, one could argue that our former control condition always depicted people in situations, where one person was in some need and where social interactions with other people were potentially looming for the main character. Therefore, we included a second control condition in the form of a video that did not depict any social interaction among humans and from which participants could not infer any goal. Consequently, we tested our hypotheses with focus on the goal condition vs. control condition 2. Second, since SVO was measured right before the manipulation in both studies, it is possible that SVO itself primed participants to behave in a proself or prosocial manner in the voluntary task (Study 1) and in the ZPG (Study 2) and thus hindered the effects of the goal manipulation. To address this issue, we conceptualized Study 3 as a two-part online study and measured SVO 1 week before the actual goal manipulation. As dependent measure, we relied on the voluntary task from Study 1. The study was designed in Unipark (Questback GmbH, 2017).

### Participants and Sample Size

Employing a second control condition video clip, we were striving for a small effect between this clip and the experimental condition. Hence, we calculated the sample size with G*Power, using *a priori* multiple regression, α = 0.05, 1 − β = 0.80, one tested predictor (SVO interacting with Dummy 1 for goal conditions vs. control condition 2), a total of five predictors (SVO, Dummy 1, Dummy 2 for control 1 vs. control 2, SVO × Dummy 1, and SVO × Dummy 2), and Δ*R*^2^ = 0.023 (*f^2^* ~ 0.023) as parameters. This yielded *N* = 351 participants. The study was designed as online study and a link was sent to the student population of the University of Graz, distributed *via* Facebook messages and Facebook groups, and finally to students which we had approached on campus and in different introductory lectures. As incentive, students were given the opportunity to take part in a lottery for vouchers.

A total of 1,148 people clicked on the link for the first part of the study and 616 completed this first part. One week later, a link to the second part was sent to these participants *via* email. A total of 642 clicked on the second link (26 clicked on it twice) of which a total of 423 reached the end of Study 3. We matched participants across study parts using anonymous ID codes and excluded those who did not fulfill our registered criteria (see https://osf.io/57ukg/). These criteria included that they should not take more than 10 min to reach the voluntary helping task after the goal manipulation, reported having not been distracted during the task, reported having had no trouble with the video buffering, and answered at least two questions correctly in the attention checks (see next section). Thus, our final sample had 371 participants, consisting mainly of students from several schools of the University of Graz. Their mean age was 22.48 years (*SD* = 3.79) and a majority of them were women (*n* = 272).

### Methods and Procedure

In the first part of Study 3, we only collected data about participants’ SVO, measured *via* Murphy et al.’s (2011) scale. Participants then provided their email address so that we could send them the link to the second part after 1 week. This second link led them to the actual experiment, in which participants were randomly assigned to one out of three conditions and watched one of three video clips. Two clips (goal condition, control 1) were identical to the ones used in Study 2. The third clip represented another, potentially more minimal control condition (control 2), showing a blue whale in the ocean (similar control condition clips with nature documentary contents were used by Schnall et al., 2010).

After three attention check questions, participants were partially debriefed and given the opportunity to volunteer in another student’s extra task that would not be monetarily rewarded. We used participants’ response to this helping behavior request as our first DV in a logistic regression model. Moreover, we used the number of pages participants voluntarily completed from this additional task as our second DV in an OLS regression model (material was adopted from Study 1). Before students could take part in the lottery, they had to answer three *goal inference* questions identical to those of Study 2 (in goal and control 1 only). Also, they were asked if they were distracted while doing the study (from 1 “not at all” to 4 “very much”) and whether they could watch the video without problems (from 1 “yes, without problems” to 4 “no, not at all”).

### Results and Discussion

#### Confirmatory Analysis

We first performed logistic regressions to evaluate participants’ initial indication to help the student as a dichotomous measure with SVO and two dummy variables (goal vs. control 2 and control 1 vs. control 2) for the three experimental conditions in Model 1, and the interactions between SVO and the conditions as additional predictors in Model 2. Model 2’s interaction terms were not significant, goal × SVO: Wald’s χ^2^(2) = 0.209, *p* = 0.647, *OR* = 1.01, control 1 × SVO: Wald’s χ^2^(2) = 1.946, *p* = 0.163, *OR* = 1.03. In Model 1, we did not find any effect of SVO, Wald’s χ^2^(1) = 0.042, *p* = 0.837, *OR* = 1.002, but we observed a significant difference between the goal condition and control condition 2, Wald’s χ^2^(1) = 3.878, *p* = 0.049, *OR* = 0.594, as well as between the control 1 and control 2 conditions, Wald’s χ^2^(1) = 7.148, *p* = 0.008, *OR* = 0.493. Contrary to our expectations, the effect of the manipulation indicated that participants who watched the control 2 clip with the whale were more willing to help (*n* = 99, 71%) than participants who watched the goal clip (*n* = 69, 59%) or the control 1 clip (*n* = 62, 54%).

**Figure 5:**
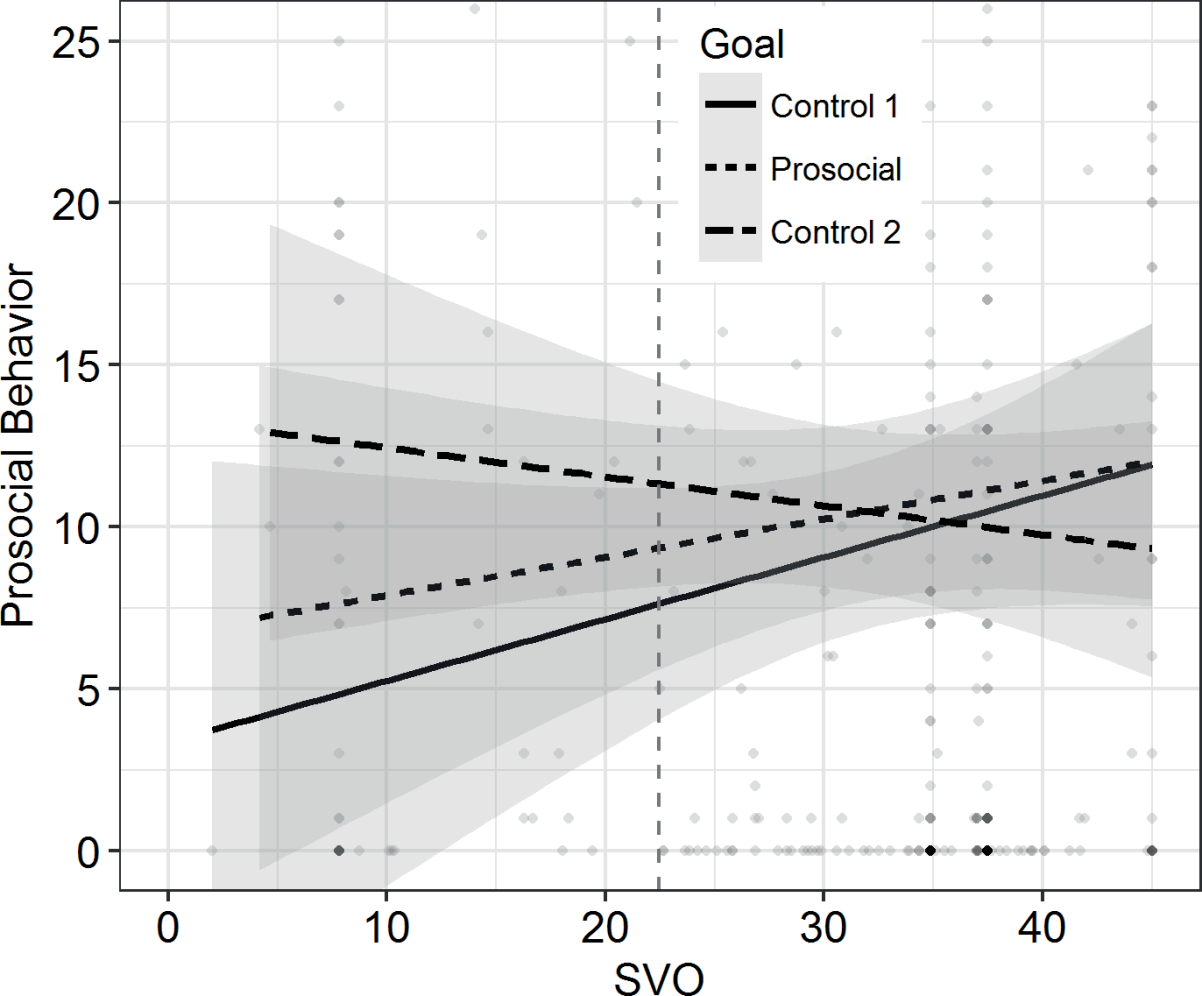
Prosocial Behavior as a function of SVO in Study 3. Separate lines signify conditions. Shades depict 95% CIs. Higher density of the scatter signifies higher concentration of data. Vertical dashed line separates proselfs (left) from prosocials (right).

However, this latter effect was not confirmed in the OLS regression with the amount of completed pages as continuous DV. There was no effect found for neither of the two dummy variables (reference group: control condition 2), goal condition: *b* = 0.181, *t*(367) = 0.102, *p* = 0.919; control 1: *b* = −1.189, *t*(367) = −0.668, *p* = 0.505, nor for SVO on casual helping, *b* = 0.052, *t*(367) = 0.898, *p* = 0.370. Here again, the model with the two interaction terms did not explain further variance, *F*(2, 363) = 0.968, *p* = 0.381, Δ*R*^2^ = 0.004; goal × SVO: *b* = 0.049, *t*(365) = 0.338, *p* = 0.735; control 1 × SVO: *b* = 0.167, *t*(365) = 1.301, *p* = 0.194. Yet, the slope of SVO in control condition 1 reached statistical significance, *b* = 0.156, *t*(365) = 2.000, *p* = 0.047, whereas the slope in control condition 2, *b* = −0.111, *t*(365) = −0.112, *p* = 0.911, and the goal condition, *b* = 0.037, *t*(365) = 0.368, *p* = 0.713, did not. Inspection of the graphs (see Figure 5) revealed that proselfs’ helping behavior in the prosocial goal condition laid in between the helping behavior of proselfs in the two control conditions. However, as people showed more prosocial values, the scores of helping behavior reached similar levels across conditions. This is clearly not in line with our initial hypothesis, as we assumed higher (and not equal) scores on prosocial behavior in the prosocial goal condition compared to control condition 2.

#### Exploratory Mediation Analysis for Goal Inference

We tested the same mediation model as in Study 2 with the goal condition (vs. control 1) as IV, goal inference as mediator, prosocial behavior as DV, and SVO as moderator on the direct and the indirect paths leading to prosocial behavior. We computed goal inference scores by adding the prosocial-related answers (coded with 1) of the three manipulation check items for the goal condition and control 1 condition. The more prosocial people are, the more their inference of observed prosocial behavior should lead to their own prosocial behavior. However, this is not what we found in the mediation analysis: It revealed that although the path between the goal condition and goal inference was significant, *b* = 1.515, *t*(229) = 17.509, *p* < 0.001, goal inference did not interact with SVO to predict prosocial behavior in a substantial manner, *b* = −0.139, *t*(226) = 1.862, *p* = 0.064. Contrary to Study 2, the two main effect slopes of the goal condition and inference just reached significance, goal: *b* = 5.550, *t*(226) = 2.016, *p* = 0.045, inference: *b* = −2.708, *t*(226) = −2.029, *p* = 0.044, but SVO did not, *b* = 0.104, *t*(226) = 1.527, *p* = 0.128. Moreover, the marginally significant inference × SVO effect was indicative for higher scores of inference leading to *less* prosocial behavior the more prosocially oriented people were (for the full model description, see OSF). The interaction term of the direct path did likewise not reveal an effect, *b* = −0.081, *t*(226) = 0.481, *p* = 0.631.

In sum, the large sample did not confirm any of the hypotheses—neither the expected effect of the goal condition or the goal condition by SVO interaction, nor the previously found effect of SVO alone could explain prosocial helping behavior. Moreover, the mediation over explicit goal inference did not contribute to our understanding of the goal contagion effect.

## Meta-Analysis

Even though goal contagion is an established effect in the literature (Aarts et al., 2004, 2008; Custers and Aarts, 2010), we did not find any evidence for it in any of our three studies. Furthermore, results were rather mixed for the predictive power of SVO on prosocial behavior: we found marginally significant (Study 1) and significant (Study 2) SVO effects, but those effects did not replicate in Study 3.

To be able to come to a more conclusive interpretation of these results, we decided to reanalyze the effects of goal manipulation and SVO across all three studies in two random-effects meta-analyses using the R package *metafor* (Viechtbauer, 2010). To align the design of Study 3 to the design of the first two studies, we excluded all participants from control condition 2 (the whale video) in Study 3. We used partial correlation coefficients as effect sizes and extracted their bootstrap standard errors (with 10,000 iterations). Before running the analyses, we set equivalence-test margins to Δ*r* = ±0.10. In line with Cohen (1988), we reasoned that an effect, which explains less than 1% of variance, could be deemed too small to be relevant beyond experimental settings. Across studies we reached a relatively large cumulated sample (*N* = 519). However, sufficient power for the equivalence test of 1 − β > 0.80 was achieved at slightly larger bounds of Δ*r* = ±0.13 (Julious, 2004; Lakens, 2017).

**Figure 6:**
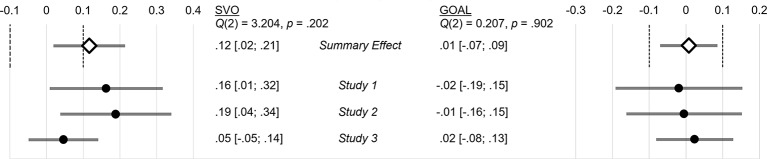
Meta-analyses for SVO and Goal (vs. control 1) on prosocial behavior; point-estimates are partial correlation coefficients controlled for Goal and SVO, respectively; 95% CIs are based on bootstrapped SEs; summary effects (◊) are based on random-effects models; vertical dashed lines are equivalence bands of Δ*r* = ±0.10, which were defined after data collection; sample sizes: *n*_S1_ = 126, *n*_S2_ = 162, *n*_S3_ = 231; control condition 2 was omitted from the analysis. Q refers to heterogeneity across studies.

Results of the meta-analyses are summarized in two forest plots in Figure 6. They show that the tiny effect of the goal manipulation, *r* = 0.01, does not differ significantly from zero and neither the upper, nor the lower bound of the 95% CI cross the equivalence bounds of Δ*r* = ±0.10. Those results do not leave much room for speculation about any potential effect of goal contagion within the scope of our studies: participants’ prosocial behavior was virtually unaffected by the manipulation material. For SVO, there is a different picture. Despite the insignificant and high-powered effect in Study 3, the summary effect of SVO, *r* = 0.12, remains outside of the equivalence-test margins and the lower bound of its 95% CI does not overlap with zero. Thus, dispositional SVO was predictive for helping behavior across studies.

## General Discussion

The investigation of determinants of prosocial behavior is a worthwhile endeavor within the psychological sciences as it has profound implications for the improvement of interpersonal relations, which could in the long run affect the societal level as whole.

Looking at the cognitive process of goal contagion—the adoption of a goal following the observation and inference of goal-directed behavior—was therefore reasonable, as previous research indicated that this phenomenon can be applied to a broad range of goals. A selection of goals encompasses earning money, casual sex, cooperative and competitive behavior, and health-conscious behaviors. Moreover, there was some limited evidence for goal contagion on prosocial behavior (Dik and Aarts, 2007).

Building onto this research, we performed three studies investigating the contagion of prosocial goals. These studies aimed at demonstrating that these kind of goals can be activated in participants when they observe someone intending to provide help to another person. Empirically, we hypothesized that participants observing a prosocial goal in another person’s behavior would be more likely to provide their help in a different kind of situation. Moreover, we tested the hypothesis that the contagion of prosocial goals is moderated by participants’ SVO. We assumed that the more prosocial participants are oriented, the stronger the goal contagion effect. Our data did not verify any of these hypotheses. In none of the three studies did we find a goal contagion effect or a moderation by SVO. In addition, exploratory mediation analyses did not reveal an influence of explicit goal inference on the adoption of the prosocial goal, either. What we observed, however, was that the more prosocially participants were oriented the more often they provided help to others.

What are possible reasons for our studies not yielding the expected effects? First, one might argue that the manipulation materials were insufficient. However, there are reasons to doubt this explanation. In Study 1, we used short vignettes, in which the main character provided help to another person in an everyday situation in the goal condition. Those texts were inspired by other goal contagion studies and were similar in design and length (Aarts et al., 2004; Jia et al., 2014). One could nevertheless criticize that text vignettes are too weak to elicit goal contagion. We therefore enhanced our manipulation material further. Specifically, we decided to produce video clips for Study 2 and 3, depicting similar everyday life helping situations as in Study 1. These videos provided vivid and rich observation of a person intending to help, which should foster a goal contagion effect. In Study 3, we further increased the impact of the manipulation by introducing a second control condition. Whereas the control conditions in Study 1 and 2 partially depicted a potential helping situation (which than resolved), the additional control video in Study 3 did not hint at any social interaction at all. In addition, we have empirical evidence in Study 2 and 3 that participants indeed inferred the goal of helping to a greater extent in the goal condition than in the control condition. Given the similarity to established goal manipulations (e.g., Dik and Aarts, 2007; Loersch et al., 2008), the vividness of the material, and the strong effect of the manipulation on explicit goal inference, it seems unlikely that an insufficient manipulation is responsible for the non-significant results.

Second, one could argue that both our DVs did not allow picking up the priming effect due to goal contagion. However, we think that the lack of a goal contagion effect in our DVs is unlikely due to the measures themselves. The DVs we employed were in line with our working definition of prosociality, emphasizing casual helping behavior, where people make a decision to volunteer without strategic concerns or fearing consequences if they do not. In Study 1 and 3, we invited participants to volunteer in an additional study conducted by a student, who could not compensate them. Such tasks in which participants are asked for voluntary help are well established (Karremans et al., 2005; Twenge et al., 2007; Greitemeyer and Osswald, 2010) and have been used repeatedly in previous goal contagion studies as DV (Aarts et al., 2004, Study 3; Dik and Aarts, 2007, Experiment 3). However, asking for help directly (even though the word “help” was not used) might undermine a potential priming effect due to goal activation^5^. Priming might work best, if the DV is applicable but ambiguous (Loersch and Payne, 2011). Thus, even though this method picked up a goal contagion effect in a previous study (Dik and Aarts, 2007), such a blatant measure might overshadow a goal contagion effect, because everybody is primed to act prosocially by the DV itself. Fortunately, this issue is addressed in Study 2, where we employed the ZPG. This game was introduced relatively recently for experimental purposes (Leiberg et al., 2011) and has also been utilized to dissect cooperative motivations and personalities (Böckler et al., 2016; but see Wilhelm et al., 2017). As participants are instructed to reach a treasure at the end of a path, the prosocial aspects of the game are more disguised than in Study 1. Furthermore, due to its flexible settings, it is one of few games enabling the measurement of spontaneous and low-cost help. Such low-cost assistance is typical for our targeted casual helping behavior, that is, situations where people usually do not worry about rewards or compensations.

Third, we think that the effects of SVO buttress the validity of our outcome variables: Although those effects were small across studies, they turned out to be robust in a small meta-analysis. This is in line with previous research, showing SVO is predictive for a diverse range of cooperative and prosocial behaviors in both experimental and real-life settings (see Bogaert et al., 2008). Hence, our studies are no exceptions.

Fourth, based on the original findings of goal contagion using similar measures (Aarts et al., 2004; Dik and Aarts, 2007; Jia et al., 2014), we were confident that the statistical power of our studies was sufficient to detect the hypothesized effect. In particular, while enhancing manipulation materials to boost the effect in theory, we increased our sample sizes successively from study to study, culminating in a large sample in Study 3. With that sample, we could have spotted a true effect of *r* = 0.15, which would have been smaller than virtually all comparable goal contagion effects reported thus far.

Besides the strengths we highlighted in the last section, there are obvious points that have to be discussed more thoroughly. A first point concerns the role of SVO. In our studies, we intentionally measured SVO first to avoid potential confounding influences by the stimulus material or the assessment of prosocial behavior. However, the concern occurred that SVO itself could have primed participants, which is why we split the assessment of SVO and the actual experiment in Study 3. SVO measurement 1 week prior to the experiment was reasonable only under the assumption that SVO is a *trait* concept. However, as research by Ackermann et al. (2016) as well as Pulford et al. (2016) implies, SVO can be sensitive to the situation. Thus, participants’ SVO during the experimental session might differ from the one measured a week earlier. This would explain to some extent why a weaker and non-significant association between SVO and prosocial behavior was found in our Study 3. *Post hoc* collection of SVO or even controlling for a shift in SVO over time could be approaches to deal with this issue in the future.

Another point for discussions might be our explicit measure of goal inference for the exploratory analyses in Study 2 and 3. We think that the depiction of a clear intention to help is a strength of our manipulation and explicit goal inference, as used in prior studies (see Aarts et al., 2004), and was employed to verify that the goal is inferred. As one would expect, we did find a strong link between the goal manipulation and explicit inference. Despite goal inference not being related to prosocial behavior, this could be interpreted as a partial support of the goal contagion model. However, according to some empirical work on the matter (e.g., Jia et al., 2014), not explicit, but implicit goal inference should mediate the goal contagion effect. In fact, an explicit attribution of a goal as the cause of another person’s behavior might be indicative for participants being too aware of the fact that helping was the other person’s goal and, hence, would fail to misattribute it as their own (Laurin, 2016). Because goal contagion is described as an automatic and unconscious process, future research might want to focus on implicit goal inference measures (Aarts et al., 2004, Hassin et al., 2005; but see Corcoran et al., 2019) to test the mediation process. Unfortunately, such a measurement could hardly be conducted after measuring the goal pursuit itself. In our research, we tried to minimize any disturbance between observing goal-directed behavior and showing goal pursuit. Therefore, we measured goal pursuit directly after the manipulation. However, we certainly agree that a systematic approach to quick, automatic, and implicit goal inference as a mediator would be beneficial and should be applied with equally powerful samples as in this present study.

### From Present to Future Research

Prosocial behavior is worth paying attention to, as it is important on societal and interpersonal levels. This research focuses on the interpersonal level and contributes to the understanding of the contagion of prosocial goals. It shows that it is difficult to assume that a straightforward and automatic cognitive process like goal contagion could foster peoples’ prosocial behavior.

For some this might not come as a surprise, because former research came to different conclusions about the determinants of prosociality. For instance, prominent lines of research often assumed rather deliberative models (e.g., Latané and Darley, 1970), involving several cognitive steps until an individual decides to provide help in certain situations to anonymous others. Other research suggested empathic concern, perspective taking, identification with the others’ goal or elevation as cognitive and emotional drivers of prosocial behavior (e.g., Batson, 1981; Haidt, 2003; Tusche et al., 2016; Michael and Székely, 2017). And even other research highlighted group norms, partly as evolutionary preferences and explained helping by the motivation to avoid punishment (Fehr and Gächter, 2002; Silk and House, 2016; Tomasello, 2016; see also Nook et al., 2016). As described in the method sections in more detail, we deliberately did not include most of those factors in our manipulation materials. We did so due to our specific interest in goal contagion and how it could uniquely elicit prosocial behavior. However, given our results one might wonder whether minimizing all those factors also minimized the likelihood of any prosocial behavior.

One possible conclusion of our research might then be that prosocial behavior is no suitable goal to be adopted following a pure goal contagion process including behavioral observation and goal inference. However, foreclosing any effect of goal contagion might be premature, as we focused only on a very specific type of prosocial behavior and it likely hinges on the mediating role of implicit goal inference, which we did not address.

As a wide array of elicited goals and moderating conditions becomes apparent in the goal contagion literature, we would recommend a meta-analytic evaluation thereof to identify generalizable differences across studies. Future research on prosocial goal contagion might then build on those findings, while taking predictors into account that showed reliable effects in other areas, such as emotions and norms (see introduction).

Regarding SVO, our final study yielded a non-significant correlation with prosocial behavior. We argue that this finding should not be overinterpreted, as it was not the confirmatory aspect of this research, the direction of the effect turned out as one would expect and the meta-analytic summary effect was in favor of SVO being predictive for casual helping as we define it. Nonetheless, the associations of SVO and casual helping and potential boundary conditions might spark interest to be pursued further.

Furthermore, other theories on prosocial traits would be worth paying attention to in order to account for more aspects of a prosocial personality in the context of the contagion of prosocial goals. Potentially, research on moral foundations (Graham et al., 2009; Haidt, 2012) and agreeableness from the Big 5 personality traits (Habashi et al., 2016) is worth mentioning here: the authors of those studies apprehend prosocial personality and traits as more complex rather than unidimensional constructs. They could also present some evidence that relations between those traits and prosocial tendencies might partly depend—yet again—on intermediary emotional processes. Thus, including these traits in future research on prosocial goal contagion could be promising.

## Conclusion

Overall, our research showed that the cognitive mechanisms of goal contagion might not be sufficient to elicit prosocial behavior in a person observing every day helping. Even though observers inferred the prosocial goal, they did not act on it when given the opportunity. For now, it remains unclear whether goal contagion is limited to specific kinds of goals—not including a prosocial goal—or whether other factors hindered the effect in our studies. However, we think that once future research has established reliable moderators and mediators within the process, a richer and more comprehensive picture can be drawn of goal contagion.

## Author Contributions

HB conducted and analyzed Study 1 and 3, analyzed Study 2, wrote the preregistration for Study 3, wrote the current version of the manuscript, and uploaded all materials and data on the OSF. AF and CF conducted Study 2 and wrote the preregistration for Study 2. UA provided substantial feedback for Study 2 and had the idea for Study 3. GK and LVE read several previous versions of the manuscript and provided feedback on all stages of the writing phase. KC provided feedback on all stages of the writing phase and gave important advice for argumentations and formulations.

### Conflict of Interest Statement

The authors declare that the research was conducted in the absence of any commercial or financial relationships that could be construed as a potential conflict of interest.

## Footnotes

^1^In the goal contagion literature the activation of goal-related words is sometimes interpreted as indicating automatic goal inference (i.e., the observer infers the goal, Jia et al., 2014) and sometimes as indicating goal adoption (i.e., the observer adopts and pursues the goal, Dik and Aarts, 2007).

^2^This is true for the Slider Measure (Murphy et al., 2011), whereas with older measures like the Triple-Dominance Measure (see Van Lange, 1999) participants are categorized directly.

^3^https://osf.io/h8zj9/

^4^https://osf.io/rt4b9/

^5^We thank a reviewer for this suggestion.
